# Pulmonary Permeability Assessed by Fluorescent-Labeled Dextran Instilled Intranasally into Mice with LPS-Induced Acute Lung Injury

**DOI:** 10.1371/journal.pone.0101925

**Published:** 2014-07-09

**Authors:** Honglei Chen, Shaoping Wu, Rong Lu, Yong-guo Zhang, Yuanyuan Zheng, Jun Sun

**Affiliations:** Department of Biochemistry, Rush University, Chicago, Illinois, United States of America; University of Illinois College of Medicine, United States of America

## Abstract

**Background:**

Several different methods have been used to assess pulmonary permeability in response to acute lung injury (ALI). However, these methods often involve complicated procedures and algorithms that are difficult to precisely control. The purpose of the current study is to establish a feasible method to evaluate alterations in lung permeability by instilling fluorescently labeled dextran (FITC-Dextran) intranasally.

**Methods/Principal Findings:**

For the mouse model of direct ALI, lipopolysaccharide (LPS) was administered intranasally. FITC-Dextran was instilled intranasally one hour before the mice were euthanized. Plasma fluorescence intensities from the LPS group were significantly higher than in the control group. To determine the reliability and reproducibility of the procedure, we also measured the lung wet-to-dry weight ratio, the protein concentration of the bronchoalveolar lavage fluid, tight and adherens junction markers and pathological changes. Consistent results were observed when the LPS group was compared with the control group. Simultaneously, we found that the concentration of plasma FITC-Dextran was LPS dose-dependent. The concentration of plasma FITC-Dextran also increased with initial intranasal FITC-Dextran doses. Furthermore, increased fluorescence intensity of plasma FITC-Dextran was found in the intraperitoneally LPS-induced ALI model.

**Conclusion/Significance:**

In conclusion, the measurement of FITC-Dextran in plasma after intranasal instillation is a simple, reliable, and reproducible method to evaluate lung permeability alterations *in vivo*. The concentration of FITC-Dextran in the plasma may be useful as a potential peripheral biomarker of ALI in experimental clinical studies.

## Background

Acute lung injury (ALI) is one of the most important causes of severe respiratory failure. It is characterized by pulmonary edema and acute inflammation in the airspaces and lung parenchyma, and it can lead to pulmonary dysfunction and potentially to death [1]. Because its pathogenesis remains ill-defined, several animal models have been used to study the pathophysiological mechanisms of ALI [2], [3]. LPS is found in the outer membrane of Gram-negative bacteria. It acts as an endotoxin, which plays a vital role in the viability of Gram-negative bacteria and induces immune and inflammatory responses in mammals [4].

In experimental mouse models of ALI, LPS administration has been shown to injure both endothelial and epithelial barriers and to cause leukocyte infiltration, which results in extravasation of the vascular fluid [3], [5]. Increased pulmonary microvascular permeability is the hallmark of ALI. Techniques to measure the permeability are therefore needed, and several methods have been reported [6], [7]. A commonly used technique for pulmonary permeability evaluation is the Evans Blue (EB) dye assay [8]. EB binds to serum albumin, and permeability changes are measured by the amount of EB-conjugated albumin (EBA) that leaks from the blood into the airways [9]. This method relies on the binding of EB to circulating albumin *in vivo* to assess protein extravasation. Although this assay has been used in numerous publications, it has not been completely standardized. The primary administration route is intravenous injection (i.v.) [6], [10], [11]. The circulation time for dye-albumin conjugated in the blood is from 10 minutes [6] to 1 hour [8]. There are also other limitations, including the surgery, the length of time involved, a complicated algorithm, and the difficulty of precisely controlling every step. Pulmonary permeability has also been measured using FITC-Dextran in the bronchoalveolar lavage fluid (BALF). This method involves the introduction of FITC-Dextran by intravenous injection and the calculation of the ratio of FITC-Dextran in the plasma and in the BALF, which requires surgery and a complicated algorithm [7], [12], [13].

An ideal technique to measure pulmonary permeability should be simple, reproducible, feasible, and of limited invasiveness. Currently, no single technique fulfills all of these criteria. The purpose of this study was to establish a new method to measure pulmonary permeability. We determined the reliability and reproducibility of FITC-Dextran instilled intranasally (i.n.) to assess alterations in lung permeability. In the present study, physiological and pathological changes in the lung were examined and correlated with the permeability and tight and adherens junction protein and mRNA expression in a mouse model of direct ALI that is induced by intranasal instillation of LPS followed by FITC-Dextran. The fluorescence intensity (FI) of plasma FITC-Dextran was measured and used as the criterion for lung permeability. Simultaneously, whether the concentration of FITC-Dextran in plasma has a dose-dependent relation with LPS, whether the concentration of FITC-Dextran in plasma via intranasal instillation of different doses of FITC-Dextran in ALI is dose-dependent, and which dose of FITC-Dextran is appropriate for lung permeability detection were also investigated and discussed in the present study.

## Results

### Histopathological changes in the lungs of LPS-induced ALI mice

**Figure 1:**
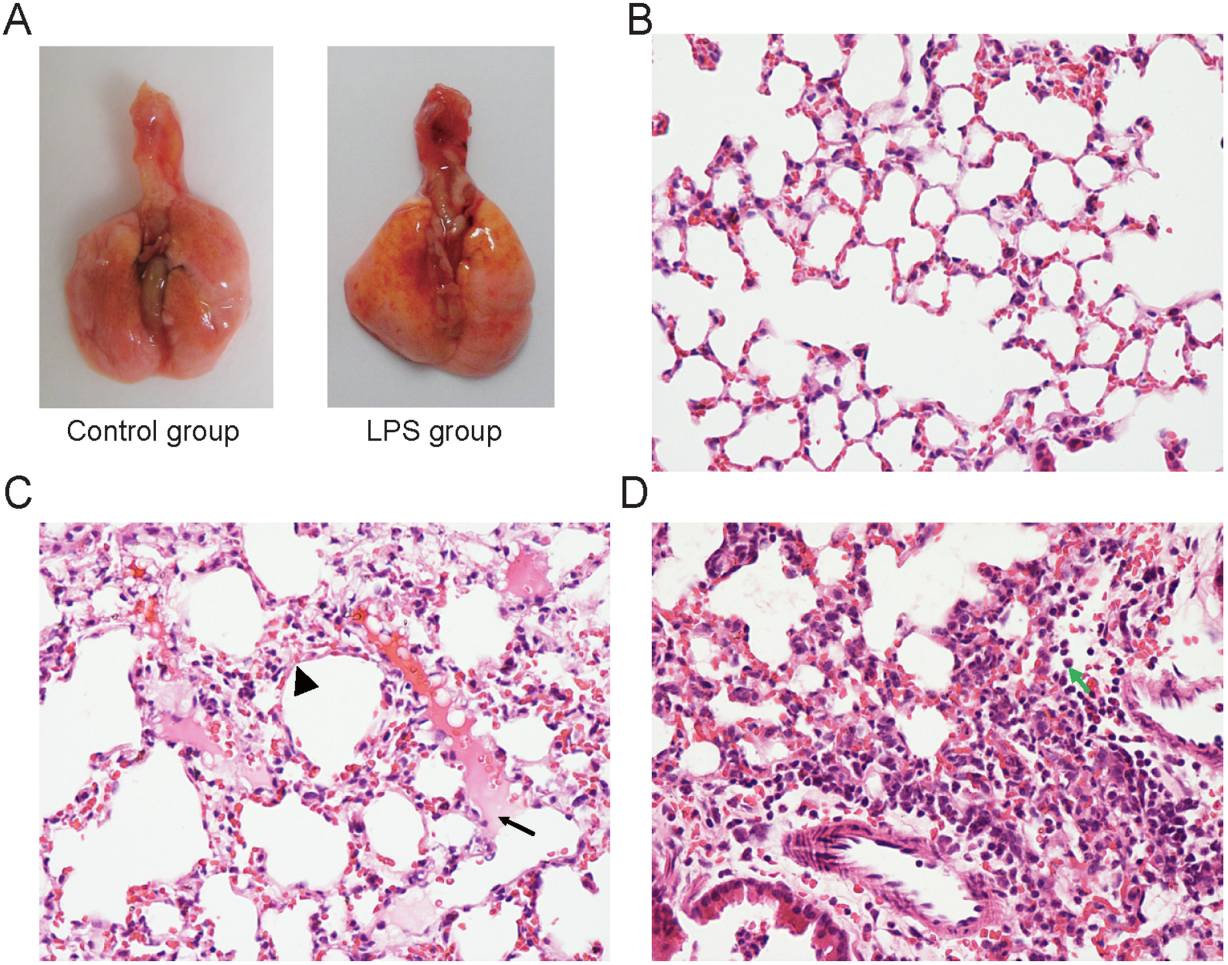
H&E staining shows pathological alterations that are characteristic of acute lung injury at 6 h after LPS instillation. *A*: The gross appearance showed yellow FITC-Dextran on the pulmonary surface after i.n. instillation with 10 mg of FITC-Dextran/kg body weight (b.w.) for 1 hour. The lungs of the LPS model group showed red dots and swelling. *B*: Representative normal lung histology. *C*: Lung edema (arrow) and alveolar wall thickening (arrow head) in the ALI mice. *D*: Infiltration of many inflammatory cells (arrow shows neutrophils) in the ALI mice induced with i.n. instillation with 0.5 mg of LPS/kg b.w. for 6 h (n = 4). B, C, D the magnification is 400X.

We first observed the gross appearance of the lungs with or without FITC-Dextran treatment. As shown in Fig. 1, yellow FITC-Dextran was observed on the pulmonary surface after i.n. instillation with 10 mg of FITC-Dextran per kg of body weight (b.w.) for 1 hour. The lungs of the LPS group (i.n., 0.5 mg/kg b.w. for 6 hours) showed red dots, swelling (Fig. 1A), and even hemorrhagic foci (Fig. S1 in File S1). Lung tissues from the control mice showed normal structures and no histopathological changes when examined under a light microscope (Fig. 1B). In contrast, lung tissues from mice that were exposed to different doses of LPS showed significant pathological changes, including lung edema, alveolar hemorrhage, alveolar wall thickening, inflammatory cell infiltration including many polymorphonuclear granulocytes (PMNs) and the destruction of epithelial and endothelial cell structure (Fig. 1C, 1D).

### Pulmonary permeability alteration in the LPS-induced ALI mice

As shown in Fig. S2 in File S1, 0.5 mg LPS/kg b.w. by i.n. instillation led to a significant increase in plasma FI compared with the control group that was instilled i.n. with 10 mg of FITC-Dextran/kg b.w. We performed a serial dilution and found that a 1∶200 dilution allowed for the detection of differences in FI between the control and LPS-treated groups (Table S1 in File S1).

**Figure 2:**
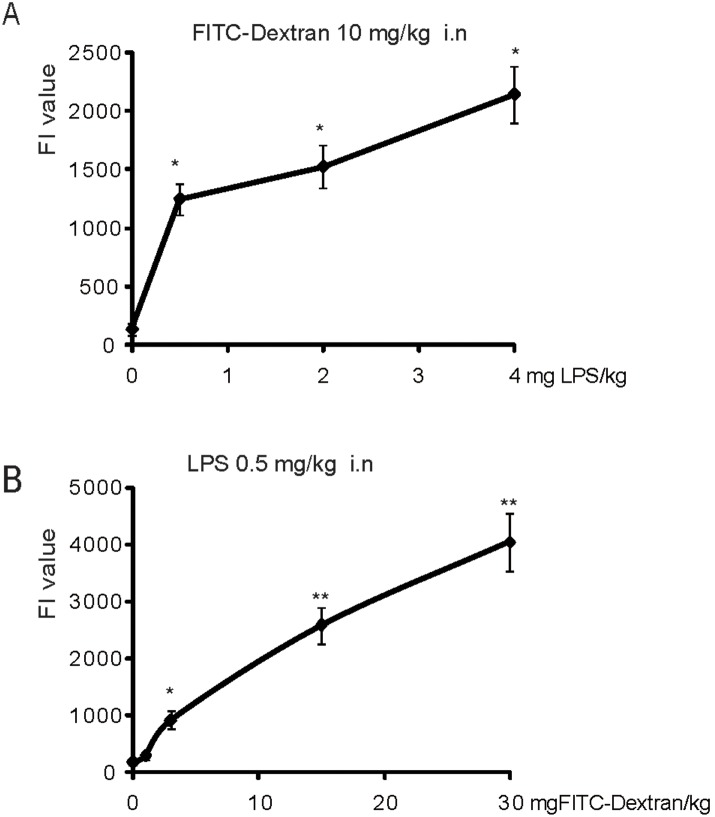
Pulmonary permeability assessed by FITC-Dextran via i.n. instillation. *A*: Lung permeability assay using 10 mg of FITC-Dextran kg b.w. via i.n. instillation in the ALI induced by different doses (0.5, 2 and 4 mg/kg b.w.) of LPS by i.n. instillation (n = 3). The fluorescence intensity (FI) of the FITC-Dextran in the plasma showed a dose-dependent increase following the different doses of LPS. *B*: Lung permeability assay using different doses (1, 3, 15 and 30 mg/kg b.w.) of FITC-Dextran via i.n. instillation in the ALI induced by 0.5 mg of LPS/kg b.w. by intranasal instillation (n = 3, *P<0.05, **P<0.01).

We further tested the plasma FITC-Dextran FI values in the ALI model induced by different LPS doses. In ALI induced by 0.5, 2 or 4 mg of LPS/kg b.w. (with intranasal instillation for 6 hours), we found that the concentration of FITC-Dextran in the plasma was increased in an LPS dose-dependent manner (Fig. 2A). Moreover, we also tested the sensitivity of the instillation of different concentrations of FITC-Dextran. The plasma FITC-Dextran was assayed in the ALI mice induced with 0.5 mg of LPS/kg b.w. for 6 h and i.n. instillation with 1, 3, 15 and 30 mg of FITC-Dextran/kg b.w. Fig. 2B shows that the concentration of plasma FITC-Dextran was elevated with the increasing initial i.n. FITC-Dextran doses. In the present study, we found that 3 mg of FITC-Dextran/kg b.w. by i.n. instillation could be sensitive enough to reflect lung permeability alteration (Fig. 2B).

**Figure 3:**
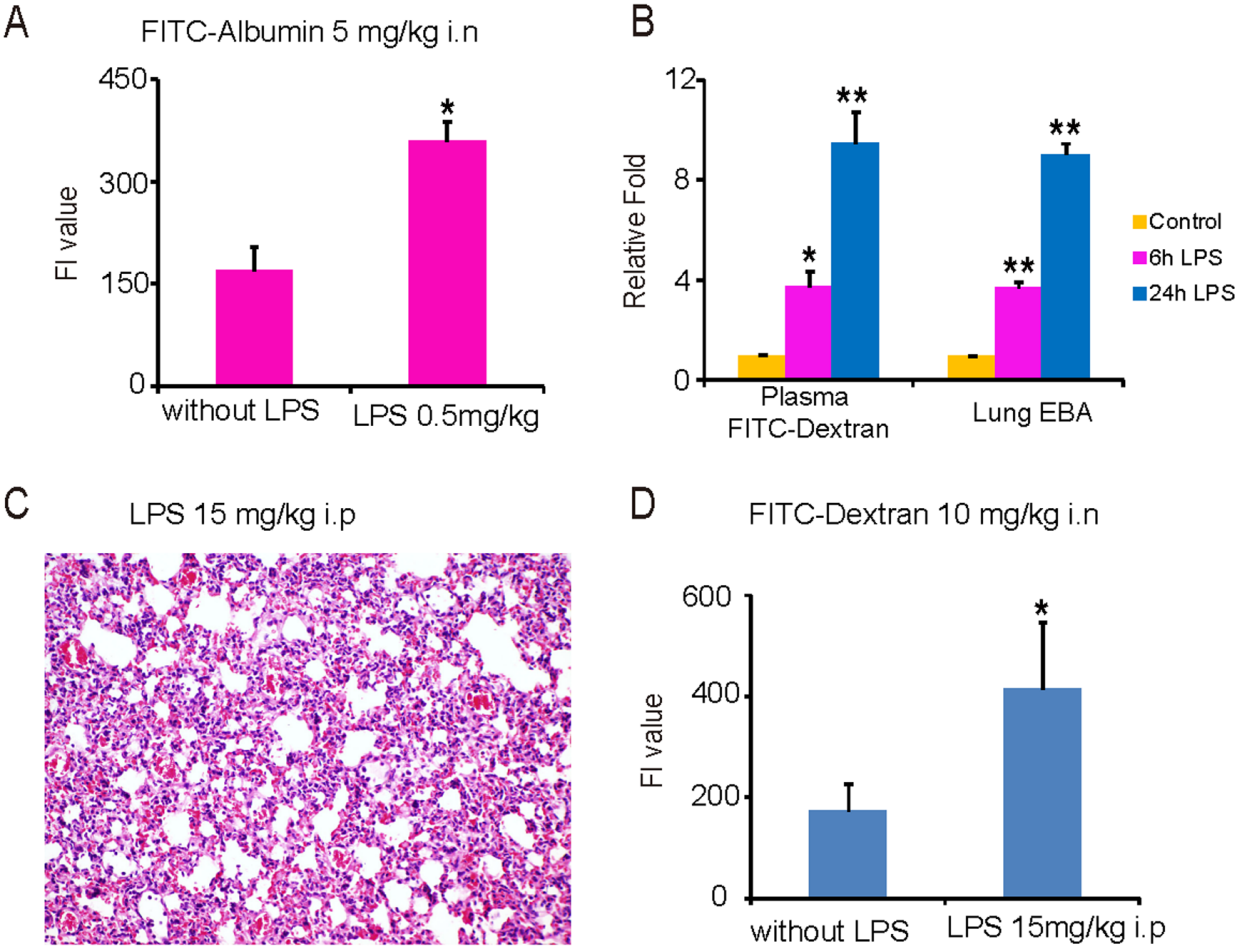
Lung permeability evaluation in the i.p. or i.n. LPS-induced ALI model by FITC-Albumin and FITC-Dextran assay. *A*: The FI value of plasma FITC-Albumin (5 mg/kg b.w., via i.n. instillation) in the ALI induced i.n. by LPS (0.5 mg/kg b.w.). *B*: Similar fold changes were found between FITC-Dextran (10 mg/kg b.w., via i.n. instillation) and EB (20 mg/kg b.w., via i.v. instillation) in the 6 h and 24 h LPS (4 mg/kg b.w., via i.n. instillation) groups compared to the control groups. *C*: Lung inflammatory lesions and thickened alveolar septa observed in ALI induced by LPS i.p. (15 mg/kg b.w.) (200X magnification). *D*: The concentration of plasma FITC-Dextran is significantly increased in the LPS i.p. group compared to the control group, with 10 mg of FITC-Dextran/kg b.w. via i.n. instillation (n = 3, *P<0.05, **P<0.01).

A parallel experiment was designed to compare the intranasal FITC-Dextran delivery approach to FITC-Albumin in LPS (i.n., 0.5 mg/kg b.w.)-induced ALI. It’s very interesting that the FI value in the plasma also could reflect lung permeability alterations with FITC-Albumin (i.n., 5 mg/kg b.w.) (Fig. 3A). Moreover, we also compared this new method to the traditional intravenous injection of EB (20 mg/kg b.w). As shown in Fig. 3B, similar increases were found with the FITC-Dextran (10 mg/kg b.w.) under i.n. instillation method and the EB i.v. method in the groups treated with LPS (i.n., 4 mg/kg b.w.) for 6 hours and 24 hours, relative to the control group.

In a different ALI model that was induced intraperitoneally (i.p.) with 15 mg of LPS/kg b.w [14] for 6 hours, we measured plasma FI after i.n. instillation of 10 mg of FITC-Dextran/kg b.w. for one hour. Lung inflammatory lesions were observed in the LPS group (Fig. 3C). The FI of the FITC-Dextran in the plasma of the LPS group was significantly increased compared to the control group (Fig. 3D).

### The lung wet-to-dry ratio, BALF protein concentration and PMN infiltration in the LPS-induced ALI Mice

**Figure 4:**
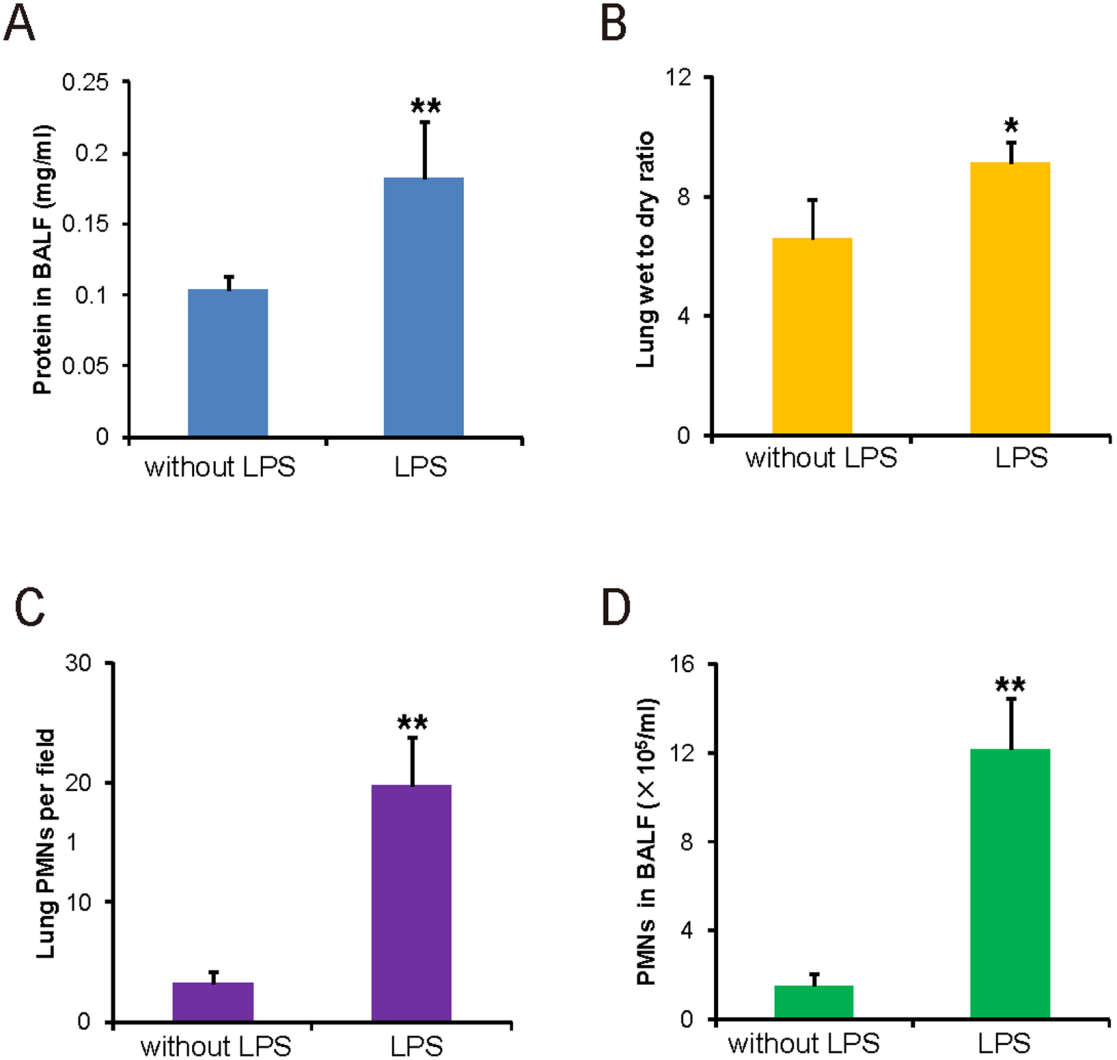
Lung permeability evaluation by traditional methods. *A*: Protein concentration in the BALF. *B*: The lung wet-to-dry weight ratios in mice with i.n. instillation with 0.5 mg of LPS/kg b.w. *C*: PMN count in the lung interstitium. *D*: PMN count in the BALF of mice with i.n. instillation with 0.5 mg of LPS/kg b.w. (n = 4, *P<0.05; **P<0.01).

The lung wet-to-dry (W/D) ratio and the total protein concentration in the BALF were evaluated at 6 hours after i.n. instillation of 0.5 mg of LPS/kg b.w. Fig. 4A and 4B show the lung W/D ratio and the total protein concentration in the BALF. As expected, the values of the LPS group were significantly higher than in the control group. LPS inhalation causes the significant recruitment of PMNs into the BALF and lung interstitium. We counted PMNs in the lung interstitium and BALF in the control and LPS groups. Figure 4C and 4D show that PMNs in the lung interstitium and BALF of the LPS group were significantly increased compared to the control group. These data indicate that the FI value of plasma FITC-Dextran was correlated with the results obtained by the traditional assays for lung permeability.

### Decrease of tight and adherens junction proteins and mRNAs in the LPS-induced ALI mice

Tight and adherens junctions are intercellular junctions that are crucial for epithelial adhesion and barrier functions in a wide variety of tissues and organs [15], [16]. A vital consequence of ALI is the disruption of the paracellular alveolar permeability barrier. The permeability barrier in terminal airspaces is mostly due to tight junctions between alveolar epithelial cells. VE-cadherin is a specific endothelial adhesion molecule that is located at the junctions between endothelial cells. VE-cadherin-mediated adhesions are vital to control vascular permeability and leukocyte extravasation [17]. Previous studies demonstrated that alveolar epithelial cells can express occludin and zona occludens 1 (ZO-1) as part of the tight junction complex [18]. In the present study, Western blots demonstrated that ZO-1, occludin and VE-cadherin protein levels were significantly decreased in the LPS group compared to the control group (Fig. S3A in File S1). As shown in Figure S3B in File S1, real-time PCR results further identified that ZO-1, occludin and VE-cadherin mRNA levels were significantly decreased in the LPS group compared to the control group. Furthermore, we detected the distribution of VE-cadherin protein by immunohistochemistry and observed that VE-cadherin was located in the cell membrane of the pulmonary vascular endothelial cells. A positive signal of VE-cadherin protein was observed in the control group, whereas a very weak signal of VE-cadherin was detected in the LPS group (Fig. S3C in File S1).

## Discussion

In the current study, we established a simple, reliable and reproducible method to assess alterations to lung permeability by instilling FITC-Dextran intranasally and then measuring its concentration in the plasma. This method was verified by using other pathological and biological methods, and the results showed that changes in the FITC-Dextran concentration in plasma were consistent with those of other assays. In parallel, we found that the concentration of FITC-Dextran in the plasma is increased in a LPS dose-dependent manner, and the concentration of plasma FITC-Dextran also increased with the initial intranasal FITC-Dextran dosage.

The pulmonary barrier has three compartments: blood, interstitium and alveolar space. ALI will damage both endothelial and epithelial cell barriers and lead to pulmonary permeability alterations. In our study, we first established a mouse model of ALI induced by LPS i.n. instillation. FITC-Dextran was instilled intranasally 1 hour before the mice were euthanized. When the FI value of the plasma FITC-Dextran was measured, our results showed that a significant difference in FITC-Dextran was detected between the LPS and control groups. Similar increases in the 6 h and 24 h LPS treatment groups relative to the control group were also identified with this new method and with traditional methods, such as intravenous injection of EB. This procedure was repeated more than ten times and confirmed that it is simple, feasible, and noninvasive, in contrast to published methods [7], [12], [13] that involve intravenous injection. We also found that i.n. instillation of 3 mg of FITC-Dextran/kg b.w. is appropriate for the assessment of alterations in lung permeability.

In this study, a parallel experiment was designed to compare intranasal FITC-Dextran delivery to the use of FITC-Albumin. As expectedly, the FI values of plasma FITC-Albumin also can reflect alterations to lung permeability. Our data suggest that FITC-Albumin via i.n. instillation functions is similar to FITC-Dextran. The intranasal FITC-Dextran delivery method for assaying pulmonary permeability will be feasible for different ALI models induced i.p. by LPS. Moreover, we evaluated the W/D ratio of the lung, the total protein concentration of the BALF and the PMN count of the lung interstitium and the BALF in the ALI mice. Significant increases in these parameters were observed in comparison to the control group. To determine whether the levels of tight and adherens junction proteins were also affected in the tissues of mice with LPS-induced ALI, Western blotting was performed with lung homogenates. The results showed that the ZO-1, occludin and VE-cadherin proteins were significantly decreased in the LPS group compared to the control group, which further indicates that pulmonary structure integrity is impaired by LPS. The real-time PCR measurements of ZO-1, occludin and VE-cadherin mRNA levels were also consistent with the alterations to the protein levels.

In summary, this study established an improved method to assess pulmonary permeability that is altered by airway vascular endothelial barrier dysfunction. The new method involves the measurement of FITC-Dextran in the plasma after intranasal instillation. The results are consistent with the W/D ratio of the lung, the protein concentration in the BALF and the PMN count of the lung interstitium and the BALF. The results are also correlated with alterations to the tight and adherens junctions that control cell permeability. Despite the significant progress that has been made in the last decade in the search for biomarkers in clinical ALI, few biomarkers can be used to predict the progression from the ‘at risk’ state or for diagnosis, evaluation of response to treatment, risk stratification or prognosis [19]. The FITC-Dextran concentration in the plasma may be regarded as a potential peripheral biomarker of ALI and may be used for experimental clinical studies.

## Materials and Methods

### Ethics statement

All animal work was approved by the Rush University Medical Center Committee on Animal Resources.

### Animals

Female C57BL/6 mice (9–11 weeks old) were purchased from Taconic (Hudson, NY). The mice were housed in microisolator cages and received food and water *ad libitum*. The laboratory temperature was 24±1°C, and the relative humidity was 40–80%. Before experimentation, the mice were allowed to adapt to the experimental environment for a minimum of one to two weeks. FITC-Dextran was purchased from Invitrogen (Catalog number: D-3305; NY, USA) or Sigma (Catalog number: 46944; St. Louis, MO, USA). Although the FITC-Dextran from Sigma had a lower titer and smaller FI values at the same dilution in comparison to the FITC-Dextran from Invitrogen, the changes in the FI values reflected the lung permeability in the LPS-induced ALI.

### ALI induced by LPS in mice and FITC-Dextran permeability assay

The mice were randomly divided into two groups: the control group and the LPS treatment group (n = 4). To induce ALI, the mice were anaesthetized by intraperitoneal Avertin solution (250 mg/kg b.w.). LPS (0.5 mg/kg b.w.; Klebsiella pneumonia, Sigma) in 50 µl of PBS was then introduced i.n. [3]. The mice in the control group were given 50 µl of PBS lacking LPS by i.n. instillation. All of the mice were euthanized six hours after LPS or PBS instillation.

Permeability alterations after LPS instillation were measured by FITC-Dextran leakage from the airways into the blood. Five hours after the LPS or PBS instillation, 50 µL of FITC-Dextran (10 mg/kg b.w.) dissolved in sterile PBS was introduced i.n. into the airways. Using protein gel loading tips, 25 µl of the FITC-Dextran solution was pipetted slowly but steadily onto each nostril of the mouse, which was held flat in a researcher’s hand. If the mouse did not inhale the fluid, pipetting was stopped until the fluid had been inhaled completely. After instillation, the mouse was set vertically for 2 min until it breathed steadily. The mice were sacrificed one hour later, and blood was collected by cardiac puncture. This procedure is recommended for the terminal stage of a study in which a single, large volume of good quality blood must be collected from experimental animals [20]. This procedure was performed as previously described [21]. Briefly, the mouse hair was degermed with 70% ethanol, and the thoracic cavity was opened with sterile forceps and surgical scissors. A 22-gauge needle that was attached to a 1-ml syringe was inserted through the apex into the heart. Negative pressure was gently applied to the syringe plunger. When blood appeared in the syringe, the plunger was gently pulled back to obtain the maximum amount of blood. Finally, the blood was treated with 10 µl of EDTA (60 mg/ml) and centrifuged at 7000 rpm for 10 min. Approximately 500 µl of plasma was harvested from each mouse. The FI of the plasma FITC-Dextran was determined at an excitation wavelength of 485 nm and an emission wavelength of 528 nm using a Synergy H1 plate reader (BioTek). Experiments were repeated three times.

### Evaluation of lung permeability by FITC-Dextran intranasal instillation in comparison to EB intravenous injection

EB intravenous injection is a well-established method for measuring lung vascular permeability. We compared this new method with the EBA assay after intravenous injection of EB. Eighteen mice were divided into three groups, which included the control and 6 h and 24 h model groups. The mice of the model groups were instilled i.n. with 4 mg of LPS/kg b.w., and the mice of the control group were instilled i.n. with 50 µl of PBS. One hour before the mice were sacrificed, 10 mg of FITC-Dextran/kg b.w. was instilled i.n. into the airway of the model and control groups. The FI of the plasma FITC-Dextran was measured as described above.

EB (20 mg/kg b.w.; Sigma-Aldrich, St Louis, MO, USA) was injected into the retroorbital venous sinus in the model and control mice for 30 minutes as previously reported [6], [10] before all of the mice were euthanized. The lungs were perfused free of blood (perfusion pressure of 5 mmHg) with PBS containing 2 mM EDTA via thoracotomy. The right lung was homogenized in PBS (1 ml/100 µg tissue), incubated with 2 volumes of formamide (18 hours at 60°C), and centrifuged at 5,000×g for 30 minutes. The optical density of the supernatant was determined spectrophotometrically at 620 nm and 740 nm using a Synergy H1 plate reader (BioTek). The extravasated EBA concentration in the lung homogenate was calculated against a standard curve (micrograms of Evans Blue dye per lung). The following formula was used to correct the optical densities for contamination with heme pigments: E620(corrected) = E620(raw)−(1.426×E740(raw)+0.030). Given the different routes of administration, the fold changes relative to the basal and not the absolute values were compared.

### Mouse ALI model induced by different doses of LPS by intranasal instillation and lung permeability assay

The mice were randomly divided into two groups: the control and LPS treatment groups. The LPS group was further divided into three subgroups, which were given 0.5, 2 or 4 mg of LPS/kg b.w. that was dissolved in sterile PBS. As described above, ALI was induced i.n. with the different doses of LPS. Five hours after the LPS or PBS instillation, 10 mg of FITC-Dextran/kg was instilled i.n. into the airways. The details of this method are the same as those described above. Six hours after LPS or PBS instillation, all of the mice were euthanized. The FI of the plasma FITC-Dextran (1∶70 dilution using PBS) in each group was determined at an excitation wavelength of 485 nm and an emission wavelength of 528 nm using a Synergy H1 plate reader (BioTek).

### Lung permeability assay with different doses of FITC-Dextran in the ALI mice

Twenty-four mice were divided into two groups, which included the control and model groups. Each subgroup had 3 mice. Four subgroups that were used as model groups were instilled i.n. with 0.5 mg of LPS/kg b.w. Another four subgroups that were used as control groups were instilled i.n. with 50 µl of PBS. Five hours after the LPS or PBS instillation, 1, 3, 15 or 30 mg of FITC-Dextran/kg b.w. was instilled i.n. into the mice. The FI of the plasma FITC-Dextran was measured using a Synergy H1 plate reader (BioTek).

### Lung permeability assay by intranasal delivery FITC-Albumin in the ALI mice

Six mice were divided into two groups, which included the control and model groups. The model group (n = 3) was instilled i.n. with 0.5 mg of LPS/kg b.w. The control group (n = 3) was instilled i.n. with 50 µl of PBS. Five hours after the LPS or PBS instillation, 5 mg of FITC-Albumin/kg b.w. (Catalog number: A9771; Sigma-Aldrich, St. Louis, MO, USA) was instilled i.n. into the airway of the model and control groups. The FI of the plasma FITC-Albumin was measured as described above.

### Mice ALI model induced by LPS intraperitoneal instillation and lung permeability assay

Six mice were divided into two groups, which included the control and the model groups. The model group was induced i.p. with 15 mg of LPS/kg b.w., and the control group was instilled i.n. with 50 µl of PBS. Five hours after the LPS or PBS instillation, 10 mg of FITC-Dextran/kg b.w. (dissolved in sterile PBS) was instilled i.n. into the airways. The details of the method are the same as those described above. The FI of the plasma FITC-Dextran (1∶70 dilution using PBS) was determined at an excitation wavelength of 485 nm and an emission wavelength of 528 nm using a Synergy H1 plate reader (BioTek).

### Lung wet-to-dry weight ratio

After i.n. instillation of LPS for 6 h, mice were euthanized. The diaphragmatic lobe of the right lung was excised separately and rapidly weighed to obtain the “wet” weight. Samples were oven dried (65°C) for 48 h to determine the stable dry lung weight. The ratio of the wet-to-dry (W/D) lung weight was calculated to assess lung tissue edema.

### Bronchoalveolar lavage fluid collection and protein determination

An increased protein concentration in the BALF can reflect an increase in capillary leakage. Immediately after death, the left lung of each mouse was ligated, and the other lung was washed by an intratracheal injection of PBS that was followed by gentle aspiration. The recovered fluid was processed to determine the protein concentration. Cells were removed by centrifugation (7,000 rpm for 10 min), and the cell-free supernatant was assayed for protein by the Bradford assay (Bio-Rad).

### Histopathologic evaluation

Histopathologic evaluations were performed on mice that were not subjected to the BALF collection. One-third of the left lung was harvested 6 h after the administration of LPS or PBS and fixed in 4% paraformaldehyde for 24 h, embedded in paraffin, stained with hematoxylin-eosin (H&E) and observed by light microscopy.

### PMN count in the lung interstitium and the BALF

The PMN count in the lung interstitium was determined by light microscopy using H&E staining and by selecting five high power fields in each case. The number of PMNs in the BALF was estimated by counting PMNs in five high power fields in each case, using the microscope slide smear technique combined with Giemsa staining. All analyses were performed in a blinded fashion.

### Statistical analysis

All values were expressed as means±SEM. Statistical analyses were performed using Student’s *t*-test for two groups and by ANOVA followed by Tukey’s test for multiple groups. A p-value of 0.05 or less was considered to be statistically significant. All of the data were analyzed using GraphPad Prism 5.

## Supporting Information

File S1 **Supporting figures and table. Figure S1,** Gross appearance of the acute lung injury induced by different doses of LPS. **Figure S2,** Lung permeability evaluation in the ALI model induced by LPS (0.5 mg/kg) via i.n. instillation. **Figure S3,** Alteration in tight and adherens junctions in the ALI induced by LPS (0.5 mg/kg bodyweight via i.n. instillation). **Table S1,** Fluorescence intensity values of different dilutions of plasma FITC-Dextran after FITC-Dextran via intranasal instillation in the ALI mice induced by 0.5 mg of LPS/kg LPS (means±SEM).
(DOCX)
