# Supplementary material for: Pulmonary Permeability Assessed by Fluorescent-Labeled Dextran Instilled Intranasally into Mice with LPS-Induced Acute Lung Injury
Source: PLoS One. 2014 Jul 9;9(7):e101925. doi: 10.1371/journal.pone.0101925 (PMC4090205; doi:10.1371/journal.pone.0101925)
Supplement: File S1 — Supporting figures and table. Figure S1, Gross appearance of the acute lung injury induced by different doses of LPS. Figure S2, Lung permeability evaluation in the ALI model induced by LPS (0.5 mg/kg) via i.n. instillation. Figure S3, Alteration in tight and adherens junctions in the ALI induced by LPS (0.5 mg/kg bodyweight via i.n. instillation). Table S1, Fluorescence intensity values of different dilutions of plasma FITC-Dextran after FITC-Dextran via intranasal instillation in the ALI mice induced by 0.5 mg of LPS/kg LPS (means±SEM). (DOCX) [file pone.0101925.s001.docx]

**Supplementary data**

**Materials and methods:**

**Detection of tight and adherens junction proteins by Western blots**

One third of the left lung tissue was harvested and cut into pieces with surgical scissors, lysed in lysis buffer, sonicated, and the protein concentration was measured. The detailed method is described in our previous study [[1](#_ENREF_1)]. The following antibodies were used: anti-zona occludens 1 (ZO-1), anti-Occludin (1:1000; Invitrogen, NY, USA), anti-VE-cadherin (1:1000; Biolegend, CA, USA) and anti-β-actin (Sigma-Aldrich, Milwaukee, WI, USA). They were visualized by ECL.

**Detection of tight and adherens junction by real time quantitative PCR**

Total RNA was extracted from one third of left lung tissue using TRIzol reagent (Invitrogen, Carlsbad, CA). The detailed method is described in our previous study[[2](#_ENREF_2)]. RNA reverse transcription was made by the iScript cDNA synthesis kit (Bio-Rad, Hercules, CA) according to the manufacturer’s directions. The RT-cDNA reaction products were subjected to quantitative real-time PCR using the MyiQ singlecolor real-time PCR detection system (Bio-Rad) and iQ SYBR green supermix (Bio-Rad). All expression levels were normalized to β-actin levels of the same sample. Relative expression was calculated as the ratio of the normalized value of each sample to that of the control mouse. All real-time PCR reactions were performed in triplicate. PCR primers were designed using Lasergene software (DNAStar, Madison, WI), the following primers were used:

ZO-1, forward 5’-AGGTCTTCGCAGCTCCAAGA -GAAA-3’ and reverse 5’-ATCTGGCTCCTCTCTTGCCAACTT-3’.

Occludin, forward 5’-AGCAGCCCTCAGGTGACTGTTATT-3’ and reverse 5’-ACGACGTTAACTCCTG -AACCAGCA-3’. VE-cadherin, forward 5’- AGAGTCCATCGCAG- AGTC-3’ and reverse 5’-CAGCCAGCATCTTGAACC-3’.

**VE-cadherin protein detection by immunohistochemistry**

Four μm thick deparaffined sections were hydrated and antigen retrieval was performed by microwave oven, sections were immersed in EDTA Buffer (1mM EDTA, 0.05% Tween 20, pH 8.0) for 15 min at 95°C and cooled for 30 min at room temperature. Sections were incubated in 3% hydrogen peroxide, followed by incubation with normal goat serum (diluted 1:100, Vector Laboratories, Inc. CA, USA). Sections were incubated with primary rat anti-mouse VE-cadherin antibody (1:100, Biolegend, CA, USA) at 4°C overnight. After washing with PBS, sections were incubated in biotinylated goat anti-rat IgG (1:300, Vector Laboratories, Inc. CA, USA ) for 30 min at 37°C, washed again with PBS, and incubated with VECTASTAIN^®^ Elite^®^ ABC kit (Vector Laboratories, Inc. CA, USA) for 30 min at 37°C. Sections were then incubated with fresh NovaRED^TM^ (Vector Laboratories, Inc. CA, USA) solution at room temperature and monitored under the microscope. After rinsing with tap water, section counterstaining was performed with hematoxylin. Sections were allowed to air dry before mounting, and observed under Nikon E600 light microscope equipped with Olympus CCD DP72. Positive cells of VE-cadherin were brown-red, localized in the cell membrane and the cytoplasm.

**Supplementary figures and table**


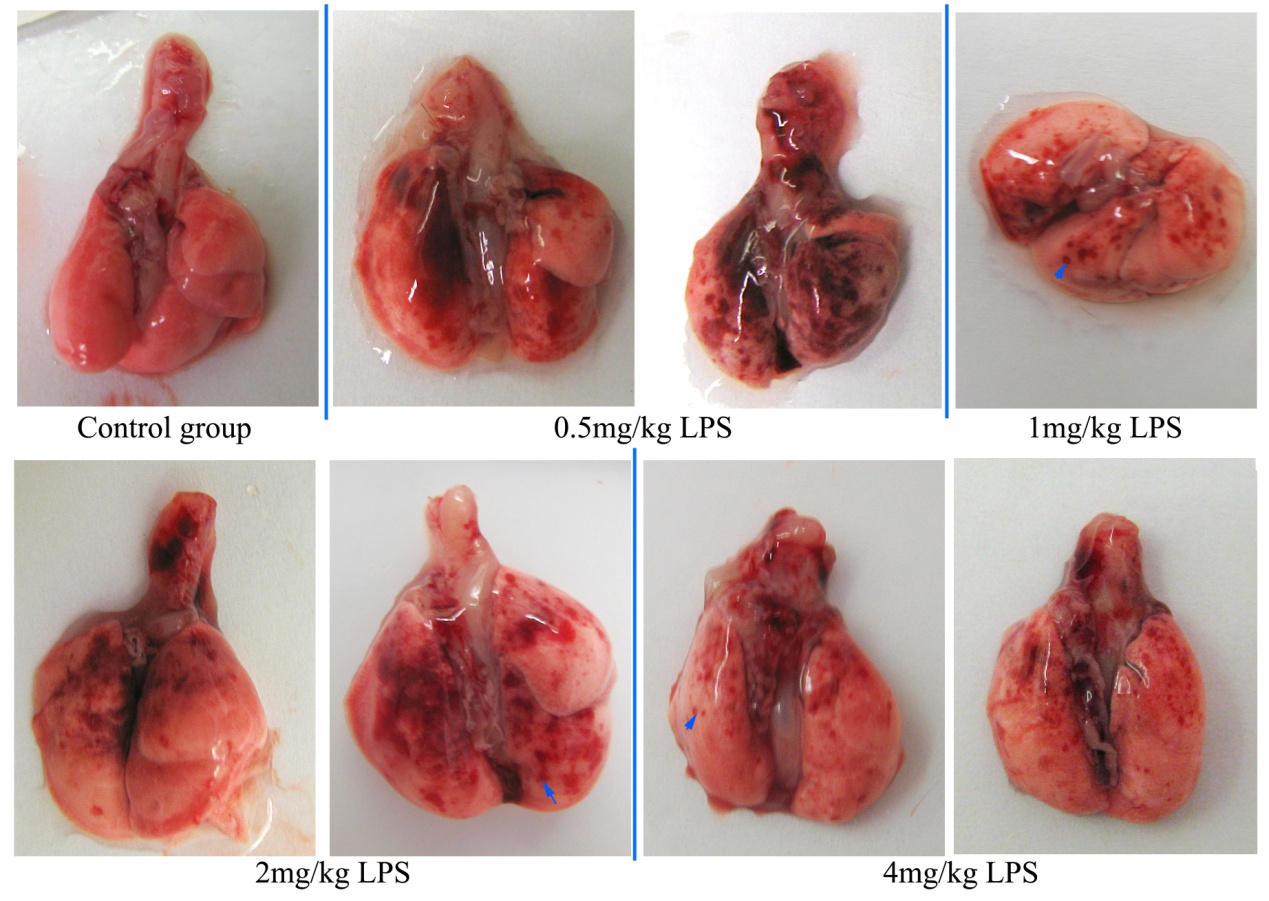


**Figure S1**. **Gross appearance of the acute lung injury induced by different doses of LPS.** Many hemorrhagic foci (blue arrow showed) can be observed in the LPS groups. ALI can be induced intranasally by all four doses.


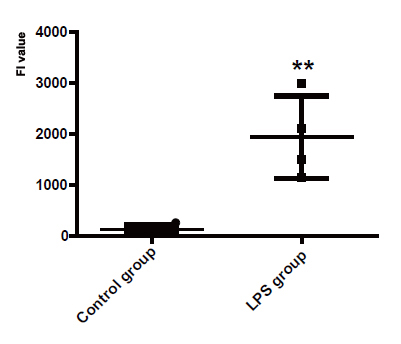


**Figure S2**. **Lung permeability evaluation in the ALI model induced by LPS (0.5 mg/kg) via i.n. instillation.** Plasma fluorescence intensity (FI) values of the LPS group were significantly higher than in the control group following i.n. instillation with 10 mg of FITC-Dextran/kg b.w. (n=4. **: P<0.01).


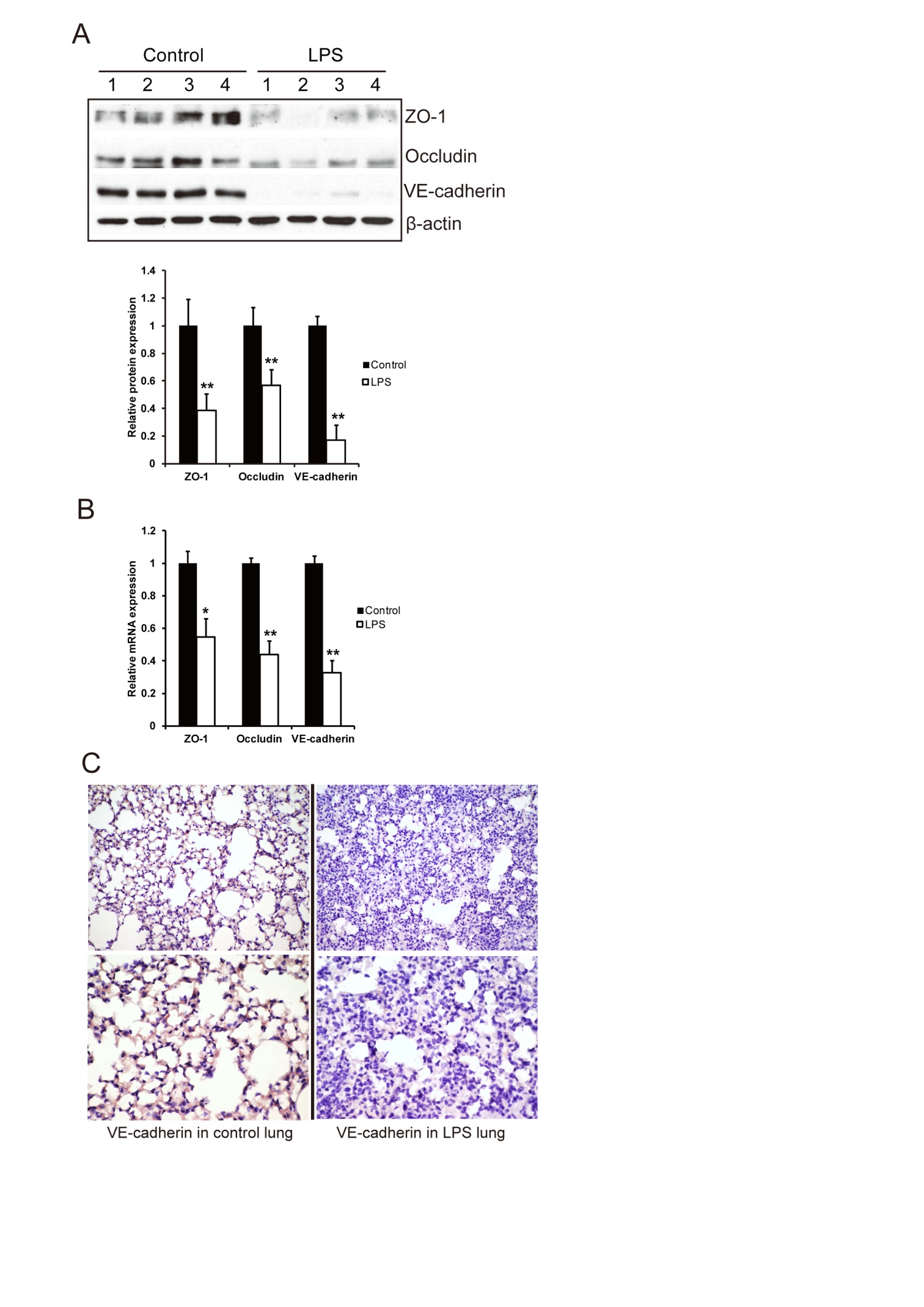

**Figure S3. Alteration in tight and adherens junctions in the ALI induced by LPS (0.5 mg/kg bodyweight via i.n. instillation).** A: LPS significantly decreased the expression levels of the ZO-1, occludin and VE-cadherin proteins. B: the ZO-1, occludin and VE-cadherin mRNA levels were decreased in the LPS group compared to the control group (n=4, * P<0.05, ** P<0.01). C: VE-cadherin was located in the cell membrane of pulmonary vascular endothelial cells. Positive signals (brown-red) of the VE-cadherin protein were observed in the control group, but a very weak signal of the VE-cadherin protein was detected in the LPS group (original magnification, upper row 200X, lower row 400X).

**Table S1** Fluorescence intensity values of different dilutions of plasma FITC-Dextran after FITC-Dextran via intranasal instillation in the ALI mice induced by 0.5 mg of LPS/kg LPS (means±SEM)**.**

| FITC-Dextran | Control group | | | LPS group (0.5 mg/kg) | | |
| --- | --- | --- | --- | --- | --- | --- |
| dilution | Mouse 1 | Mouse 2 | Mouse 3 | Mouse 1 | Mouse 2 | Mouse 3 |
| 50 | 787.3±59.1 | 871.3±44.3 | 3651.3±155.9 | 10149.7±256.6 | 8820.3±244.4 | 18679.7±204.3 |
| 100 | 414.7±59.2 | 456±45.2 | 1820±132.3 | 5481.7±195.5 | 5014±229.7 | 10261.7±178.7 |
| 200 | 252.7±13.7 | 273.7±42.6 | 1058±85.1 | 2804.3±115.5 | 2674.3±161.7 | 5436±73.7 |
| 400 | 130.3±13.3 | 175±11.8 | 559±42.5 | 1436±62.5 | 1355±97.7 | 2749.7±142.5 |
| 800 | 62.3±19.1 | 84.7±20.6 | 241.3±21.4 | 828.7±51.4 | 784.7±57.5 | 1401±84.6 |
| 1600 | 30.7±8.5 | 41.7±10.6 | 107±14.4 | 526.7±31.4 | 477.7±34.8 | 779.7±26.9 |
| 3200 | 14.7±7.23 | 18.3±6.8 | 50±7.5 | 277.7±24.8 | 272±36.3 | 451±44.8 |
| 6400 | 0 | 0 | 22±6.9 | 139±38.1 | 117±32.2 | 188.3±33.7 |
| 12800 |  |  |  | 62±8.5 | 55.5±10.6 | 91±9.9 |

**References:**

1. Liao AP, Petrof EO, Kuppireddi S, Zhao Y, Xia Y, et al. (2008) *Salmonella* type III effector AvrA stabilizes cell tight junctions to inhibit inflammation in intestinal epithelial cells. PLoS One 3: e2369.

2. Zhang YG, Wu S, Xia Y, Chen D, Petrof EO, et al. (2012) Axin1 prevents *Salmonella* invasiveness and inflammatory response in intestinal epithelial cells. PLoS One 7: e34942.
